# Fluorescent Peptide Biosensor for Probing the Relative Abundance of Cyclin-Dependent Kinases in Living Cells

**DOI:** 10.1371/journal.pone.0026555

**Published:** 2011-10-18

**Authors:** Laetitia Kurzawa, Morgan Pellerano, J. B. Coppolani, May C. Morris

**Affiliations:** Centre de Recherches en Biochimie Macromoléculaire, CRBM-CNRS-UMR 5237, Université de Montpellier, Department of Molecular Biophysics and Therapeutics, Montpellier, France; Roswell Park Cancer Institute, United States of America

## Abstract

Cyclin-dependant kinases play a central role in coordinating cell growth and division, and in sustaining proliferation of cancer cells, thereby constituting attractive pharmacological targets. However, there are no direct means of assessing their relative abundance in living cells, current approaches being limited to antigenic and proteomic analysis of fixed cells. In order to probe the relative abundance of these kinases directly in living cells, we have developed a fluorescent peptide biosensor with biligand affinity for CDKs and cyclins in vitro, that retains endogenous CDK/cyclin complexes from cell extracts, and that bears an environmentally-sensitive probe, whose fluorescence increases in a sensitive fashion upon recognition of its targets. CDKSENS was introduced into living cells, through complexation with the cell-penetrating carrier CADY2 and applied to assess the relative abundance of CDK/Cyclins through fluorescence imaging and ratiometric quantification. This peptide biosensor technology affords direct and sensitive readout of CDK/cyclin complex levels, and reports on differences in complex formation when tampering with a single CDK or cyclin. CDKSENS further allows for detection of differences between different healthy and cancer cell lines, thereby enabling to distinguish cells that express high levels of these heterodimeric kinases, from cells that present decreased or defective assemblies. This fluorescent biosensor technology provides information on the overall status of CDK/Cyclin complexes which cannot be obtained through antigenic detection of individual subunits, in a non-invasive fashion which does not require cell fixation or extraction procedures. As such it provides promising perspectives for monitoring the response to therapeutics that affect CDK/Cyclin abundance, for cell-based drug discovery strategies and fluorescence-based cancer diagnostics.

## Introduction

Cell cycle progression is driven by a family of Cyclin-Dependent Kinases (CDKs), serine/threonine protein kinases whose sequential activities promote phosphorylation of key substrates involved in cell growth and division [1]–[4]. CDK/cyclin complexes are formed through association of a catalytic CDK with a regulatory cyclin partner, which plays a major role in promoting activation of the CDK by inducing significant conformational changes, in defining substrate specificity, and in targeting the heterodimeric complex to well-defined subcellular locations [5]–[8]. Formation of functional CDK/cyclin complexes is conditioned by expression of either counterpart, although the lack of expression of one CDK or cyclin may be compensated through formation of complexes which do not normally occur, as has been reported in knockout mice lacking CDK4, CDK6 or CDK2,CDK4,CDK6, so as to ensure essential proliferative functions [9], [10].

CDK/cyclin levels and activities are frequently altered in human cancers, thereby contributing to sustain aberrant proliferation in cancer cells [11], [12]. A subset of mutations of CDK4 and CDK6 are known to confer a selective growth advantage through loss of natural inhibitor (CKI) binding, whilst other mutations have been reported to promote CDK1, CDK2 or CDK4 overexpression [13], . In addition gene amplification, protein overexpression, mislocalization or expression of truncated variants of cyclins which are associated with aberrant CDK activity have been reported in a wide range of cancers including breast, ovarian, prostate, colorectal and lung cancer, lymphoma, myeloma and sarcoma [15]–[19]. Despite the oncological relevance, prognostic value and pharmacological attractivity of CDK/Cyclins, there are no direct means of assessing their relative abundance in living cells. Indeed, the development of non-invasive sensing technologies to probe these biomarkers is largely limited by their intracellular localization. As such detection of CDKs and Cyclins remains essentially limited to classical antigenic approaches following cell or tissue fixation procedures, or to identification of circulating autoantibodies reporting on them as tumour-associated antigens from sera of cancer patients [20];yet the information obtained remains limited to individual subunits, as are no tools that report on the status of the biologically relevant CDK/Cyclin complexes. This not only restricts fundamental studies of physiological or pathological signals that modulate expression and assembly of CDK/Cyclin complexes in living cells, it also refrains development of diagnostic approaches and of strategies to assist therapeutic programmes by monitoring response to anticancer drugs.

In this article we report on the design and characterization of a fluorescent peptide biosensor, whose fluorescence increases in a sensitive fashion upon recognition of CDK/Cyclins, and which retains endogenous CDK/Cyclin complexes from cell extracts. CDKSENS biosensor was further applied to assess the relative abundance of these kinases in living cells, through fluorescence imaging and ratiometric quantification, following facilitated delivery by a non-covalent cell-penetrating peptide. We show that this fluorescent peptide biosensor/carrier strategy allows to detect subtle differences in CDK/Cyclin levels between different cell lines, or when tampering with the levels of a single CDK or cyclin, in a standardized and sensitive, yet non-invasive fashion. CDKSENS is the first biosensor to afford direct readout of CDK/Cyclin levels in living cells, providing a unique opportunity to identify cells in which these complexes are overexpressed, or on the contrary present defective assemblies, thereby offering promising perspectives for cancer diagnostics, for monitoring response to therapeutics, and for cell-based drug discovery strategies.

## Results

### Design and in vitro characterization of a CDK/Cyclin peptide biosensor

**Figure 1:**
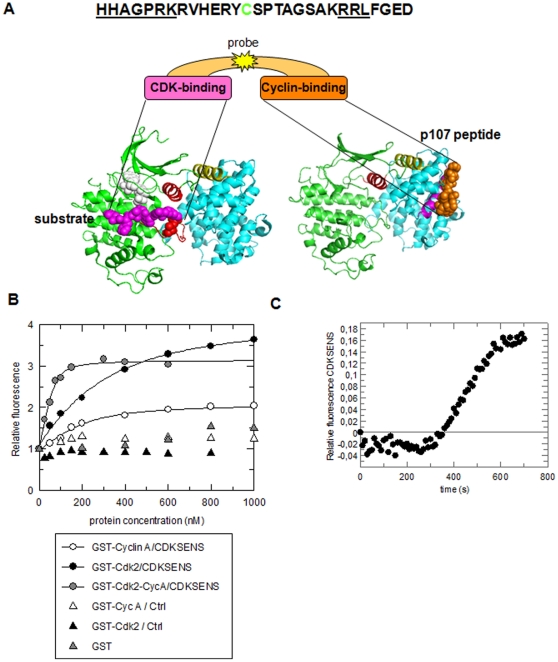
Design and in vitro characterization of CDKSENS. **A.** Sequence and schematic representation of CDKSENS together with the crystal structures of CDK2/cyclin A/HHASPRK peptide substrate (PDB 1QMZ) and CDK2/cyclin A/p107 RRLFGE peptide (PDB 1H28). CDK2 is shown in green, the PSTAIRE helix in red, and the peptide substrate in magenta; cyclin A is in cyan with the alpha 5 helix in yellow and the p107 peptide in orange **B.**Titration of 200 nM CDKSENS-FITC and Ctrl-FITC peptide with recombinant CDKs, cyclins, preformed CDK/cyclin complexes, GST or MBP. Changes in fluorescence are shown relative to the initial fluorescence of CDKSENS (or Ctrl) in the absence of target **C.** Kinetics of 200 nM CDKSENS response to a fixed concentration of CDK2/Cyclin A. Changes in fluorescence over time are relative to CDKSENS fluorescence in the absence of CDK2/Cyclin A.

In order to develop a means of probing CDK/cyclin complexes both in vitro and in living cells, we resolved to design a fluorescent biosensor that would report on the relative levels of these complexes with high selectivity and sensitivity. To this aim, we designed a 29mer peptide biosensor, CDKSENS, to recognize molecular interfaces specific to CDK-cyclin complexes, which could be labelled with a synthetic fluorescent probe whose spectral properties would be affected upon docking of the biosensor onto CDK/cyclin complexes. Since canonical substrate sequences alone are fairly unspecific for cyclin-dependent kinases, CDKSENS was deliberately designed as a biligand, one moiety intended to bind the CDK, the other the cyclin. Most CDK-cyclin substrates show strong dependency on the cooperative recognition of a consensus S/TP phosphorylation site by the CDK and a distal RXL cyclin-binding motif that is recruited by a shallow, hydrophobic patch known as the cyclin groove, which enhances overall substrate affinity and catalytic efficiency [21]–[23]. CDKSENS was therefore designed to bear a CDK-binding moiety derived from the CDC6 substrate of CDK2 [23] in which the phophorylatable serine residue was substituted by a glycine to avoid phosphotransfer from the CDK, HHAGPRK, and a cyclin-binding moiety bearing the RXL recruitment sequence derived from the retinoblastoma pocket protein p107, RRLFGE [24]. Since the RXL substrate recognition site in the cyclin is 40 Å from the catalytic site of the CDK, they were joined by a 15mer linker derived from the natural sequence of p107, immediately upstream of the cyclin-binding motif; the first serine within this sequence was replaced by a cysteine, thereby providing an unique accessible thiol for coupling of a fluorescent probe at a central position between the CDK- and the Cyclin-binding motifs RVHERYCSPTAGSAK. A schematic representation of CDKSENS is shown in **Fig. 1A**, together with the 3D models of CDK2/cyclinA/substrate peptide and CDK2/cyclinA/p107 peptide.

In order to establish the validity of this design, we first asked whether CDKSENS could interact with its targets in vitro. To this aim, it was labelled on its internal cysteine with FITC, and titrated with purified, recombinant forms of CDKs and cyclins. Binding of CDK and Cyclin partners resulted in significant enhancement in CDKSENS fluorescence with a 3–4 fold increase (300–400%) at saturation (**Fig. 1B**). When CDKSENS docks onto CDK/cyclin complexes, the fluorescent probe is introduced into an entirely different environment from its free form in solution, and is therefore subject to changes in solvent polarity which directly affect its spectral properties, a process known as solvatochromism [25]. FITC is particularly sensitive to changes in its environment, and this is also the case for cyanine probes such as Cy3 and Cy5 (**Fig. S1A**), but not for Alexa probes, which when coupled to CDKSENS did not yield any significant fluorescence enhancement upon titration with CDK/Cyclins (data not shown). Hence the choice of an environmentally-sensitive fluorescent probe is essential for CDKSENS to report on the presence of CDK/Cyclin complexes. Dissociation constants determined from the titration curves revealed that CDKSENS exhibits high affinity for both CDKs and cyclins, in the 100–200 nM and 60–70 nM range, respectively, with the exception of cyclin B, whose affinity was 7–8-fold lower than other cyclins, consistent with the presence of two mutations close to its cyclin-recruitment patch (Glu220 to Gln211 and Tyr280 to Phe271) previously reported to reduce affinity for RXL substrates [26] (**Table 1** **)**. We further found that CDKSENS biosensor presents significantly higher affinity for the CDK2-cyclinA complex than for monomeric CDK2 or cyclin A, with a dissociation constant value of 8 nM+/−5 nM. CDKSENS presented an equally high affinity for CDK1-cyclinB, CDK2-cyclinB and CDK1-cyclin A complexes, with dissociation constant values of 6+/−2 nM, 9+/−4 nM, 18+/−6 nM respectively **(** **Table 1** **and Fig. S1B)**. Taken together, these data indicate that the choice of the fairly conserved CDK- and Cyclin-binding sequences allows CDKSENS to bind all CDKs and cyclins, and clearly demonstrate that its biligand structure ensures preferential recognition of CDK/cyclin complexes over monomeric CDKs and cyclins. In contrast, titration with GST or MBP did not affect the fluorescence of CDKSENS-FITC, inferring there was no unspecific binding. Likewise, FITC-labelled control peptides derived from the substrate sequence alone, or from an irrelevant control peptide, lacking both the consensus substrate sequence and the RXL motif (Ctrl peptide: VESSDTIDNVKSKIQDKEGC) did not recognize recombinant CDKs or cyclins. Finally, having assessed the sensitivity and affinity of CDKSENS for its targets, we examined the kinetics of CDKSENS response to a fixed concentration of target. This experiment revealed that CDKSENS responds fairly rapidly to CDK2/CyclinA, the first signs of fluorescence enhancement appearing after a lag of 350 s (6 min) and reaching complete enhancement after 600 s (10 min) **(** **Fig. 1C** **)**.

**Table 1:** Dissociation constant values and fluorescence enhancement of CDKSENS-FITC upon titration with recombinant CDKs, cyclins or preformed CDK/cyclin complexes.

Recombinant Target	Kd (nM)	Fluorescence enhancement (fold)
GST-CDK1	114+/−42	4.4
GST-CDK2	193+/−33	4
GST-Cyclin A	71+/−18	2.1
MBP-Cyclin B1	467+/−256	3
GST-Cyclin E	58+/−23	2.4
GST-CDK2/Cyclin A	8+/−5	3.1
GST-CDK1/Cyclin B	6+/−2	2.5
GST-CDK2/Cyclin B	9+/−4	2.5
GST-CDK1/Cyclin A	18+/−6	2.4
GST	no binding	no variation
MBP	no binding	no variation
Ctrl/GST-CDK2	no binding	no variation
Ctrl/GST-Cyclin A	no binding	no variation

### CDKSENS binds endogenous CDK/Cyclin complexes from cell extracts

**Figure 2:**
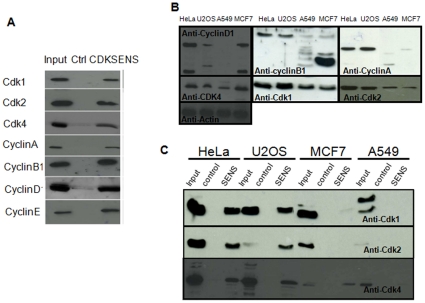
CDKSENS binds CDK/Cyclin complexes from cell extracts. **A**, Pulldown experiments performed with immobilized CDKSENS or Ctrl peptide (Ctrl) and from HeLa cell extracts reveal that all CDKs and Cyclins are retained specifically by CDKSENS, not by Ctrl peptide. **B**,Western blots of CDKs and Cyclins in normalized total extracts from HeLa, U20S, MCF-7 and A549 cells, as indicated **C**, Pulldown experiments performed as described in (A) with the cell lines described in (B); CDKSENS (SENS) Ctrl peptide (control); Western blotting for CDK1, CDK2 and CDK4.

To further evaluate the ability of CDKSENS to recognize endogenous CDK/cyclin complexes from cell extracts, we performed pulldown experiments following immobilization of CDKSENS on CNBr Sepharose. These experiments revealed that in HeLa cells, in which all CDKs and Cyclins are well expressed, all are equally well retained by CDKSENS, but not by the Ctrl peptide which lacks all relevant CDK and Cyclin binding sequences (**Fig. 2A**). Likewise, pulldown experiments performed with the osteosarcoma cell line U20S showed that CDKSENS efficiently retained all CDKs and cyclins (**Fig. 2B and C**). However pulldowns performed with cell lines in which cyclins were not expressed at wild-type levels, or as full-length proteins, such as lung cancer A549 and breast cancer MCF-7 cells, revealed that the corresponding CDK partner was not retained by CDKSENS (**Fig. 2B and C**). For instance CDKSENS retained CDK1 and CDK2 complexes from both HeLa and U20S cells, but not from MCF7 or A549 cells, which both lack wildtype levels of cyclin A and B. CDK4 complexes were retained by CDKSENS from HeLa, U20S and MCF7 cells, but not from A549 cells, which do not express detectable levels of D cyclins. These data indicate that CDKSENS does not recognize monomeric CDKs in cell extracts, but requires the presence of cyclins to recognize stable CDK/cyclin complexes, in agreement with the preferential affinity of CDKSENS for recombinant CDK/Cyclin complexes over monomeric subunits reported in **Fig. 1** **and** **Table 1**.

### Cellular Internalization and characterization of the CDK/Cyclin peptide biosensor

**Figure 3:**
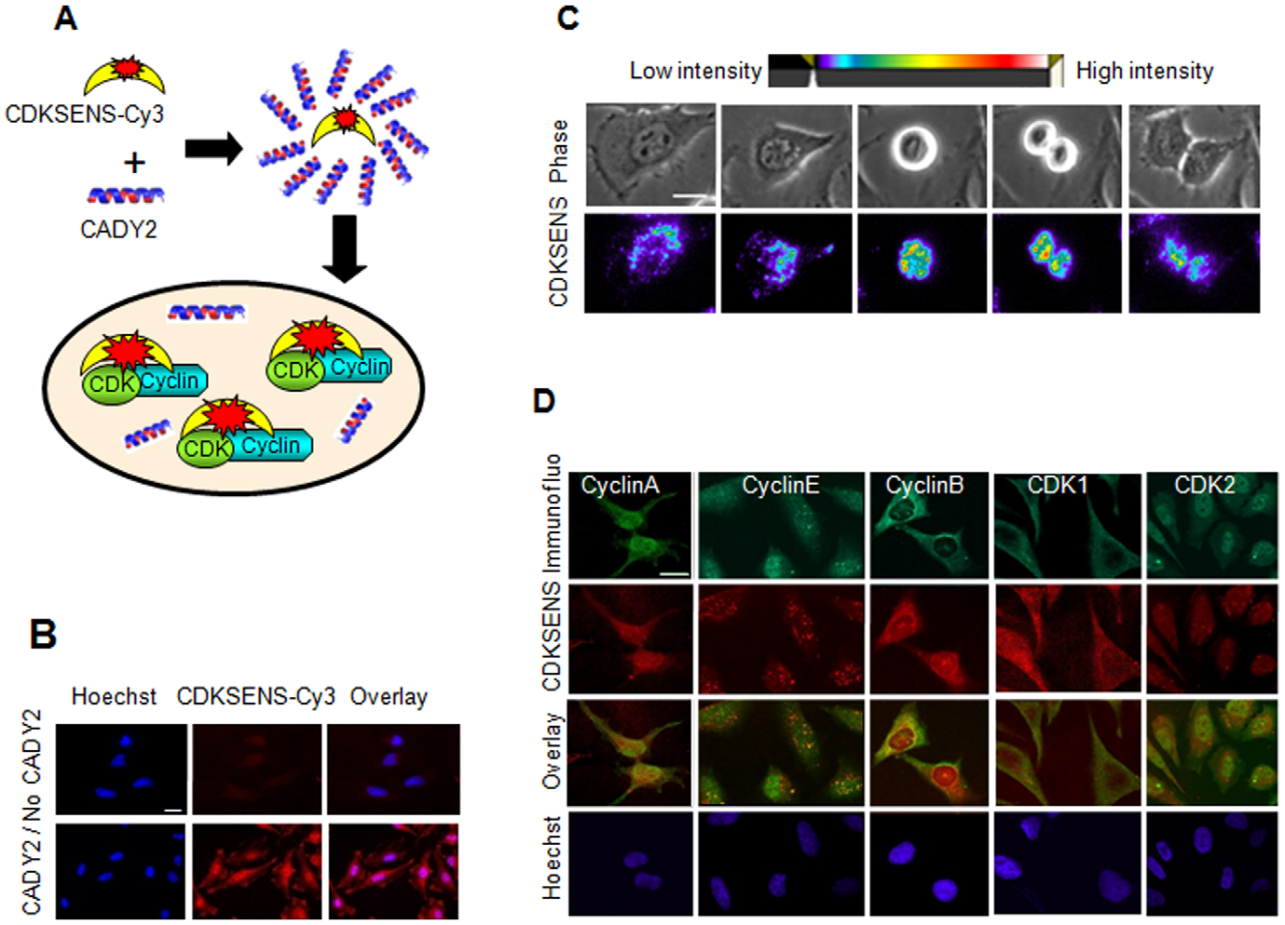
CADY-mediated delivery and characterization of CDKSENS subcellular localization. **A**, Schematic representation of CDKSENS complexation with CADY2 and delivery into cultured cells **B**, CDKSENS-Cy3 complexed with CADY2 at 40∶1 ratio is efficiently internalized by HeLa cells (lower panels) in contrats to CDKSENS-Cy3 alone (upper panels). **C**, Timelapse micrographs of dividing cells: phase contrast (upper panels) and spectral representation of relative CDKSENS-Cy3 fluorescence intensity (bottom panels). **D**, Subcellular localization of CDKSENS-Cy3 delivered into HeLa cells with CADY2, and of endogenous CDKs and Cyclins detected by indirect immunofluorescence and imaged by confocal microscopy. Bars in (B), (C) and (D) correspond to 20 um.

In order to apply CDKSENS to live-cell imaging, it was labelled with cyanine probe Cy3, which was equally sensitive in reporting on the presence of CDK/Cyclins in vitro (**Fig. S1A**). CDKSENS-Cy3 was complexed with the cell-penetrating peptide CADY2, which we have previously shown to efficiently deliver fluorescently-labelled peptides into cultured cells [27] (**Fig. 3A**). CADY2/CDKSENS-Cy3 complexes overlaid onto HeLa cells efficiently promoted cellular uptake of the fluorescent biosensor (**Fig. 3B**). Phase contrast and fluorescence time-lapse microscopy were employed to monitor changes in the distribution of CDKSENS-Cy3 fluorescence, together with changes in cell morphology through the cell cycle, revealing the dynamic distribution of the biosensor in the cytoplasm through interphase, gradually accumulating around the nucleus prior to mitosis (**Fig. 3C** **and Movie S1**). In order to determine whether the subcellular localization of CDKSENS overlapped with that of its cellular targets, HeLa cells into which CDKSENS-Cy3 had been introduced were fixed and indirect immunofluorescence was performed with antibodies directed against CDK1, CDK2, Cyclin A, Cyclin B or Cyclin E (**Fig. 3D** **and Fig. S2**). Comparison between the subcellular localization of CDKSENS-Cy3 and that of the different CDKs and cyclins by confocal microscopy did not reveal significant lack of colocalization of the fluorescent biosensor with any CDK or cyclin, in line with the ability of CDKSENS to recognize a broad range of CDK/cyclin complexes, whose differential patterns of expression, assembly and localization overlap throughout the different phases of the cell cycle.

### CDKSENS reports on the relative abundance of CDK/Cyclins in living cells

In order to apply CDKSENS to the quantification of endogenous CDK/Cyclins in living cells, independently of cell morphology, we devised a strategy based on the ratiometric quantification of CDKSENS-Cy3 fluorescence with respect to that of the Ctrl peptide labelled with Cy5, which was equally well internalized into HeLa cells with CADY2 (**Fig. S3**). Fluorescently-labelled CDKSENS and Ctrl peptide were complexed to CADY2 and introduced into living cells, and the fluorescent signal of these different species was measured directly by live-cell imaging. The ratio of CDKSENS-Cy3/Ctrl-Cy5 fluorescence was then determined on 3–4 fields of 10–15 cells and the average value was used to reflect the relative abundance of CDK/Cyclin.

**Figure 4:**
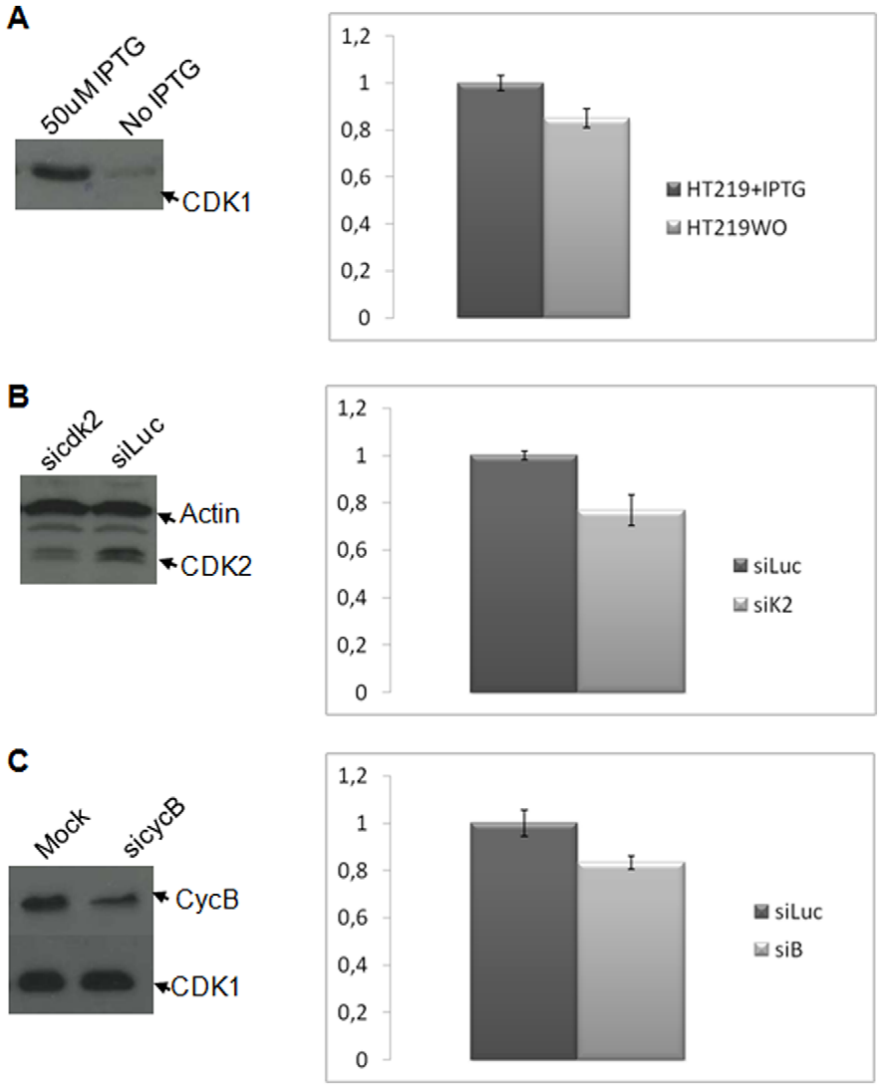
CDKSENS reports differences that affect CDK/Cyclin abundance associated with a single CDK or cyclin in cellulo. **A**, HT2-19 cells cultured with (+) IPTG or without (wo) for 4 days. **B**, HeLa cells treated with 100 nM siRNA directed against CDK2 (sicdk2) or luciferase (siLuc). **C**, HeLa cells treated with 100 nM siRNA directed against Cyclin B1 (sicycB1) or luciferase (siLuc). All left panels present Western blots of normalized cell extracts. Right panels illustrate representative examples of CDKSENS-Cy3/Ctrl-Cy5 fluorescence between treated and untreated cells, performed on 3–4 different fields of 10–15 cells. The mean calculated between the values of 3–4 different fields, is shown together with the standard deviation (**see** **Table 2**).

We first verified whether differential expression of a single CDK or cyclin was sufficient to measure quantitative differences through this approach. To this aim, we co-delivered fluorescently-labelled CDKSENS and Ctrl peptide into the HT2-19 cell line, in which CDK1 expression is under control of an IPTG-inducible promoter [28]. When these cells were grown in the absence of IPTG for 4 days, Western blotting revealed that CDK1 protein levels were significantly reduced (>80%) and consistently, CDKSENS-Cy3/Ctrl-Cy5 ratio was 20% lower in these cells, compared to cells grown with 50 uM IPTG (**Fig. 4A** **,** **Table 2** **, Table S1**).

**Table 2:** Ratiometric quantification of CDKSENS-Cy3/Ctrl-Cy5 fluorescence in HT2-19 cells induced to express CDK1 (+IPTG) or not (WO), in HeLa cells treated with siRNA targeting luciferase (siLuc), or CDK2 (siCDK2), or cyclin B (siCycB).

Fluorescence CDKSENS-Cy3/Ctrl-Cy5		Mean Ratio CDKSENS-Cy3/Ctrl-Cy5	
HT219+IPTG	WO IPTG	Mean HT219+IPTG	Mean WO IPTG
5,565739401	4,247635348	5,37+/−0,18	4,36+/−0,21
5,210906398	4,236269139	**Relative Mean Ratiometric Values**	
5,344875205	4,612407668	1+/−0,033	0.81+/−0,04
**siLuc**	**siCDK2**	**Mean siLuc**	**Mean siCDK2**
7,674257647	5,655287202	7,63+/−0,14	5,86+/−0,50
7,428888119	6,156148923	**Relative Mean Ratiometric Values**	
7,771713461	5,252854394	1+/−0,019	0.77+/−0,066
7,641928691	6,364627584		
**siLuc**	**siCycB**	**Mean siLuc**	**Mean siCycB**
6,502745388	5,837569677	6,8+/−0,38	5,66+/−0,19
6,673396111	5,682751484	**Relative Mean Ratiometric Values**	
7,226137167	5,468652038	1+/−0,055	0.83+/−0,027

In each experiment, the ratiometric value is given for 3 or 4 fields of cells (n = 10–15) and the mean ratiometric values are given together with their standard deviation. These values were then normalized to the highest mean ratiometric value.

In a second set of experiments, HeLa cells were treated siRNA targeting CDK2, leading to a partial reduction of CDK2 protein levels (50%) detected by Western blotting. Ratiometric quantification of CDKSENS-Cy3/Ctrl-Cy5, a significant decrease in the fluorescence ratio, compared to cells treated with siRNA targeting luciferase (**Fig. 4B** **,** **Table 2**), with an average difference of 20% and a very low standard deviation between independent sets of experiments, indicative of the robustness of this approach (**Table S1**). Finally, we asked whether CDKSENS could report on differences of a single cyclin, which would consequently affect the assembly of CDK/Cyclin complexes. HeLa cells treated with siRNA targeting CyclinB, yielded on average 15% lower CDKSENS-Cy3/Ctrl-Cy5 values compared to mock-treated cells, which could be associated with a 36% knockdown of CyclinB protein levels probed by Western blotting (**Fig. 4C** **,** **Table 2** **, Table S1**). Taken together, these experiments indicate that CDKSENS is sufficiently sensitive to measure differences in the relative abundance of CDK/cyclin complexes when tampering with one single CDK or cyclin subunit.

### CDKSENS reports differences in CDK/Cyclin levels between healthy and cancer cells

**Figure 5:**
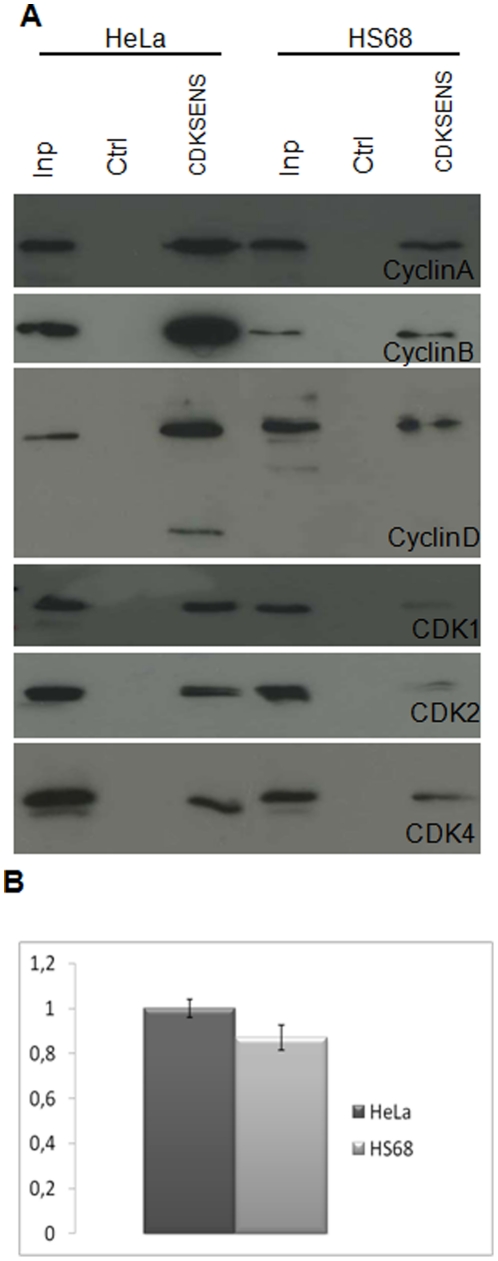
CDKSENS reports differences in the overall abundance of CDK/cyclins between HeLa cells and HS68 fibroblasts. **A**, Western blots of CDKs and Cyclins in normalized total extracts (Inp) and pulldowns from normalized HeLa or HS68 cell extracts **B**, Representative example of CDKSENS-Cy3/Ctrl-Cy5 fluorescence measured in HS68 and in HeLa cells. The mean calculated between the values of 3 different fields of 10–15 cells, is shown together with the standard deviation (**see** **Table 3**).

Since many cancer cells exhibit alterations in the relative abundance of CDKs and Cyclins, we asked whether CDKSENS could be applied to highlight differences in CDK/Cyclin levels between healthy and cancer cells. To this aim, we first chose to compare healthy human foreskin HS68 fibroblasts with the HeLa cervical cancer cell line. As shown in **Fig. 5A**, Western blotting of different CDKs and Cyclins in normalized cell extracts reveals significantly higher levels of Cyclin B, and somewhat higher levels of Cyclin A, and CDK4 in HeLa cells than in HS68 fibroblasts. Consistent with this, pulldown experiments revealed a net reduction in the levels of all CDK/Cyclin complexes retained by CDKSENS from HS68 relative to HeLa cell extracts. CDKSENS-Cy3 and Ctrl-Cy5 were co-delivered into these cell lines and their fluorescence was measured by live-cell imaging. Ratiometric quantification of CDKSENS-Cy3/Ctrl-Cy5 consistently revealed greater values for HeLa cells, with an average difference of 15% (**Fig. 5B** **,** **Table 3**).

**Table 3:** Ratiometric quantification of CDKSENS-Cy3/Ctrl-Cy5 fluorescence in HeLa, HS68, U20S, MCF7 and A549 cells.

Fluorescence CDKSENS-Cy3/Ctrl-Cy5		Mean Ratio CDKSENS-Cy3/Ctrl-Cy5	
HeLa	HS68	Mean HeLa	Mean HS68
7,20930083	6,306949025	7.21+/−0,28	6.14+/−0,35
6,928643917	6,379195062	**Relative Mean Ratiometric Values**	
7,497741061	5,744844153	1+/−0,039	0.85+/−0,048
**HeLa**	**U20S**	**Mean HeLa**	**Mean U20S**
7,462551653	6,244561064	6,79+/−0,53	7,16+/−0,65
6,964679638	7,168471954	**Relative Mean Ratiometric Values**	
6,503735882	7,702927701	1+/−0,079	1,05+/−0,095
6,247960678	7,517560305		
**HeLa**	**MCF7**	**Mean HeLa**	**Mean MCF7**
6,99509956	6,313753978	7,29+/−0,39	5,99+/−0,33
7,723968439	5,518310547	**Relative Mean Ratiometric Values**	
6,928643917	6,099266915	1+/−0,053	0.82+/−0,046
7,497741061	6,029347325		
**HeLa**	**A549**	**Mean HeLa**	**Mean A549**
6,436566914	5,578696443	6,72+/−0,37	5,44+/−0,16
7,132974124	5,257761322	**Relative Mean Ratiometric Values**	
6,589420498	5,472799273	1+/−0,054	0.81+/−0,024

In each experiment, the ratiometric value is given for 3 or 4 fields of cells (n = 10–15) and the mean ratiometric values are given together with their standard deviation. These values were then normalized to the highest mean ratiometric value.

**Figure 6:**
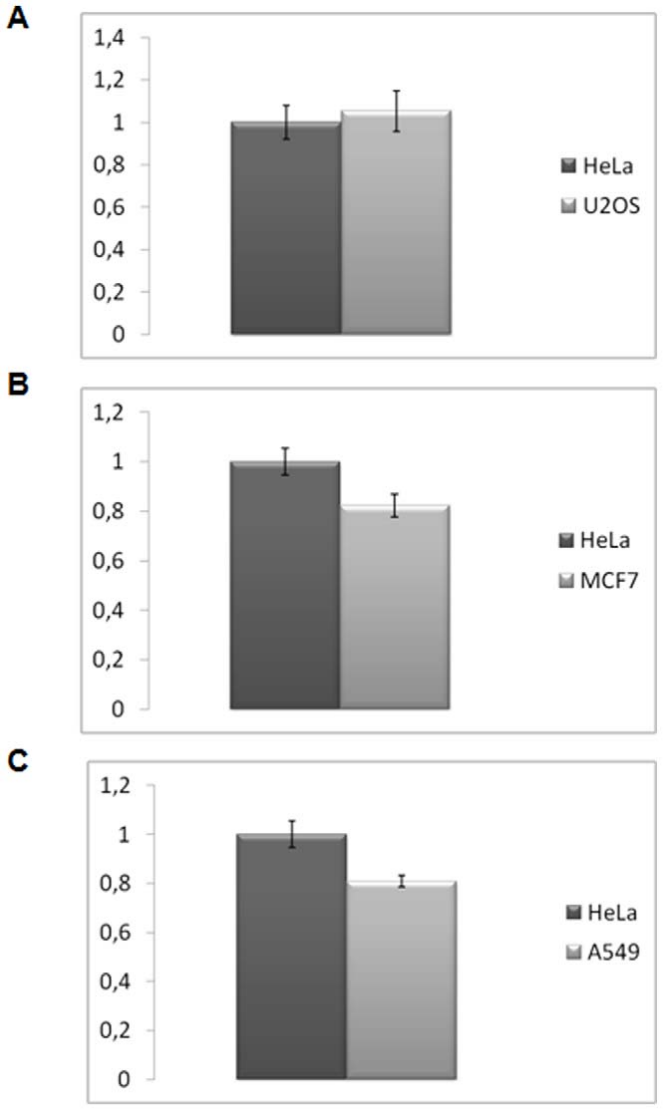
CDKSENS allows to assess the relative abundance of CDK/cyclins in different cancer cell lines. CDKSENS-Cy3/Ctrl-Cy5 fluorescence was determined between different cancer cell lines **A**, HeLa and U20S; **B**, HeLa and A549; **C**, in HeLa and MCF7. The mean ratiometric value corresponding to CDKSENS-Cy3/Ctrl-Cy5 was calculated between the values measured in 3–4 different fields of 10–15 cells, is shown together with the standard deviation (**see** **Table 3**).

We further applied CDKSENS to compare CDK/Cyclin levels between different cancer cell lines, whose features have already been described above in **Fig. 2**, using the HeLa cell line as a reference. CDKSENS-Cy3 and the Ctrl-Cy5 peptide were introduced into each of these cell lines, and the relative fluorescence of CDKSENS-Cy3 over that of Ctrl-Cy5 was determined. As reported in **Fig. 6** and **Table 3**, ratiometric quantification of CDKSENS-Cy3/Ctrl-Cy5 did not reveal a significant difference between HeLa cells and the U20S osteosarcoma cell line, in line with the Western blot of total extracts and of pulldown experiments presented in **Fig. 2**, which shows essentially identical levels of all CDKs and Cyclins and of CDK/Cyclin complexes in these two cell lines. In contrast, CDKSENS-Cy3/Ctrl-Cy5 values were consistently 18–20% lower in A549 and MCF7 cells compared to the HeLa cell line, indicative of lower relative abundance of CDK/Cyclin complexes (**Fig. 6**, **Table 3**), in agreement with the data shown in **Fig. 2**. For each of these cell lines, ratiometric values were determined from several independent sets of experiments, which revealed very similar relative values for each cell type, and reproducible differences between cell lines, indicative of the robustness of this approach (**Table S1**).

## Discussion

Together with advances in live-cell imaging technology, the development of fluorescent probes that report on enzyme levels, activities or conformations has provided new perspectives for the study of dynamic biological processes, for monitoring the cell cycle status and cell proliferation, whilst also offering new tools for diagnostics and drug discovery [29]–[32]. Whilst genetically-encoded single-chain FRET-based activity reporters have been successfully applied to probe a broad variety of enzymatic activities, combined efforts in fluorescence chemistry and chemical biology have lead to the design of an equally potent class of biosensors that incorporate small synthetic probes with unique spectral properties, such as enhanced photostability and environmental sensitivity, onto peptide and polypeptide scaffolds. Synthetic peptide and polypeptide biosensors that report on target concentration, enzymatic activity or protein conformation in complex biological samples, cell extracts and living cells, have been successfully developed, as exemplified by the LifeAct biosensor of actin dynamics [33], solvatochromic GTPase bioprobes [34], dark-quench reporters [35], chelation-enhanced bioprobes [36], and smart peptide probes that are selectively delivered into tumor cells following enzymatic activation [37], to name but a few. Aside from the latter example, one of the major drawbacks of nongenetic biosesnors is their inability to cross cell membranes, thereby requiring facilitated delivery into cells. Nonetheless, major advances in the field of peptide and protein delivery over the last twenty years have provided new strategies to circumvent this issue, in particular the use of protein transduction domains and cell-penetrating peptides, which constitute most efficient means of introducing biological molecules into living cells and in vivo [38].

In this study, we have combined the design of a fluorescent peptide biosensor, CDKSENS, that reports on the presence of CDK/Cyclin complexes through sensitive changes in fluorescence, with a non-invasive delivery strategy based on a cell-penetrating peptide which requires neither chemical coupling nor genetic fusion, thereby ensuring efficient cellular uptake and release of the bioprobe into the intracellular milieu. The peptide nature of CDKSENS makes it very easy to handle and straightforward to label and allows for a controlled and direct application both in vitro and in cellulo. Here we demonstrate that this biosensor can be used to probe the relative abundance of CDK/cyclins in vitro, in cell extracts, and in living cells in a standardized fashion through ratiometric quantification of its fluorescence over that of a control peptide.

A good biosensor should recognize and bind its target with respectable affinity and specificity, and exhibit changes in fluorescence which reflect the presence or activity of the target in a sensitive fashion, both with respect to intensity and speed. CDKSENS was designed to bear both a CDK- and a cyclin-binding moiety, in order to ensure preferential recognition of CDK/Cyclin complexes, which are biologically relevant, as opposed to monomeric CDKs and cyclins, which do not display any activity. We have shown that through its biligand design, CDKSENS biosensor preferentially binds CDK/Cyclin complexes in vitro, and that it cannot bind a CDK in the absence of its cyclin partner in cell extracts. Hence this feature ensures specific and high affinity recognition CDK/Cyclin complexes.

We have further shown that CDKSENS can be applied to probe the relative abundance of CDK/cyclins in living cells in a standardized and sensitive fashion through ratiometric quantification of its fluorescence over that of an inert control. As shown in the experiments with the HT2-19 cell line, and with siRNA targeting cyclin B or CDK2, CDKSENS is sufficiently sensitive to report on differences in the levels of a single CDK or cyclin. In this respect, it is noteworthy that a 36% decrease in the levels of cyclin B alone was consistently associated with a 15% reduction in CDKSENS-Cy3/Ctrl-Cy5 ratio, whilst changes in a single CDK yielded 20% difference in this value. We have also shown that CDKSENS distinguishes cell lines expressing high levels of CDK/Cyclins, such as HeLa and U20S cells, from cell lines expressing reduced levels of several complexes, such as HS68 fibroblasts, the breast cancer cell line MCF-7 or the lung cancer cell line A549. In contrast, when there are practically no differences between CDK and Cyclin levels between two cell lines, as for U2OS and HeLa cells, in which Western blotting indicates less than 5% difference in CyclinD levels only, ratiometric quantification of CDKSENS/Ctrl yielded essentially identical values. These data demonstrate that differences in CDKSENS/Ctrl values are truly associated with differences in the relative abundance of CDK/Cyclins, underscoring the specificity and sensitivity of this technology.

CDKSENS technology offers several advantages over strategies relying on antigenic detection. First, CDKSENS provides information on the status of CDK/Cyclin complexes which is not conveyed by antibodies that recognize individual subunits. Second, CDKSENS technology is essentially non-invasive, since it is based on live-cell fluorescence imaging and does not call for cell fixation or extraction procedures. Third, CDKSENS allows for rapid detection of cells that present differences in CDK/Cyclin levels, without discriminating between one or another complex; this feature is particularly interesting given that most cancer cells present defects in several CDKs and Cyclins.

Finally, since alterations in CDK/cyclin levels have been reported in a wide variety of cancers, and associated with negative prognosis, it is important to identify cells in which overexpression of these complexes is truly relevant for therapeutic intervention. We have shown that CDKSENS provides a unique means of assessing the relative abundance of CDK/Cyclins in living cells, in a standardized fashion, and a good indication as to whether cells express high levels of these heterodimeric kinases, or decreased or defective assemblies. These data have strong implications for diagnostic and therapeutic applications. Indeed, we have previously shown that reduction of cyclin B1 levels by siRNA or PNA administration efficiently blocks growth of tumour xenografts in mice [39], [40], and we show here that CDKSENS provides a sensitive readout of differences in cyclin B1 levels following treatment of cultured cells with cyclin B1 siRNA. Hence CDKSENS presents strong potential for monitoring response to therapeutic strategies targeting CDKs or cyclins, and offers promising perspectives for development of cancer diagnostics, and cell-based drug discovery approaches.

## Materials and Methods

### Peptide synthesis, protein expression, purification and labelling

CDKSENS and Ctrl peptides were purchased from GL Biochem, (Shanghai, China), labelled on their unique cysteine with fluorescein-isothiocyanate (FITC), Cy3- or Cy5-maleimide, and further purified on NAP-5 columns (GE Healthcare). Recombinant Glutathione-S-transferase (GST)-CDKs and cyclins were expressed in E.coli and purified by chromatography as described previously [41].

### Steady-State Fluorescence Titration Experiments

Titration experiments were performed at 25°C in a Polarstar Spectrofluorimeter (BMG Labtech). 200 nM FITC-labelled CDKSENS were titrated with purified, recombinant GST-tagged CDK1, CDK2, cyclins A, and E1, MBP-cyclin B, GST or MBP in 200 ul potassium phosphate buffer, pH 7.2, 150 mM NaCl at 25°C in 96-well microplates, and changes in fluorescence emission were recorded at 520 nm following excitation at 485 nm. Data analysis and curve fitting were performed using the GraFit Software (Erathicus Ltd) and a standard quadratic equation, as described previously [41]. It should be noted that cleaving either GST or MBP tag resulted in significant precipitation and loss of recombinant CDKs and cyclins; therefore fusion proteins were used in all assays.

#### Cell Culture, extract preparation and pulldown experiments

Cell culture media, serum and antibiotics were purchased from Invitrogen. HeLa, HS68, A549, MCF-7 and U2OS cells were obtained from ATCC and were cultured in Dulbecco's Modified Eagle Medium (DMEM)+Glutamax supplemented with 10% Fetal Calf Serum (FCS), 1 mM penicillin and 1 mM streptomycin at 37°C in an atmosphere containing 5% CO2. HT2-19 cells, provided by Dr. A.Porter, were cultured in DMEM supplemented with non essential amino acids, antibiotics, sodium pyruvate, glutamine, and 10% FCS and grown with 50 uM isopropyl-thiogalactoside (IPTG) for maximal induction of CDK1 [28]. Cell extracts were prepared in lysis buffer containing 50 mM TrisHCl, pH 7.4, 150 mM NaCl, 0.1% NP40, 0.1% Deoxycholate, 2 mM EDTA, 1 mM phenylmethlsulfonyl fluoride (PMSF), CompleteTM protease inhibitors (Roche), 50 mM NaF, 40 mM β-Glycero-phosphate, 1 mM Na3VO4 and normalized following spectrophotometric dosage at 280 nm. For pulldown experiments, CDKSENS or Ctrl peptide were crosslinked to CNBr Sepharose beads, then incubated with equal concentrations of HeLa cell extracts for 1 hr. Samples were then processed for SDS-PAGE and CDK, Cyclin retention was detected by Western blotting.

### Antibodies for Western Blotting and Indirect Immunofluorescence

Antibodies against Cyclin A (H432, sc-751), Cyclin B1 (GNS1, sc-245), Cyclin D1 (C20, sc-717), Cdk1 (C19, sc-954) Cdk2 (M2, sc-163), and Cdk4 (C22, sc-260) were purchased from Tebu-Bio (Santa-Cruz), anti-actin from Sigma (A2668), and used at 1∶1000 dilution for Western blotting, except for anti-cyclin B1 at 1∶500 dilution, 1∶100 for indirect imunofluorescence. Secondary antibodies labelled with Alexa-488 were used for indirect immunofluorescence.

### siRNA Transfections

siRNA targeting Cyclin B was a Smart Pool™ M003206-02 purchased from Dharmacon. SiRNA targeting CDK2: 5′-AAGGUGGUGGCGCUUAAGAAA-3′ and Luciferase: 5′-CUUACGCUGAGUACUUCGATT-3′ were purchased from Eurogentec. siRNA transfections were performed for 72 h with the cell-penetrating siRNA carrier CADY as described previously [42].

### Cell-penetrating peptide-mediated delivery

For cellular uptake, 3 uM CDKSENS or control peptide were complexed with the cell-penetrating peptide CADY2 at 1∶40 molar ratio in 100 ul PBS for one hour at 37°C, overlaid onto cells for 15 min, then for 45 min in the presence of DMEM, and finally complemented with 10% serum, as described previously [27].

### Microscopy

For fixed observations, cells were treated for 20 minutes with paraformaldehyde, nuclei were stained with Hoechst 33342 (Sigma), and cells were mounted with Prolong Gold AntiFade Reagent (Invitrogen). Epifluorescence images were acquired on a Leica DM6000 microscope (Leica Microsystems) piloted by the Metamorph software (Universal Imaging). For colocalization experiments, images were acquired on a Zeiss axioplan2/LSM510 META confocal microscope. Live-cell imaging was performed on a Zeiss Axiovert 200 M piloted by the Metamorph software. Images were processed with ImageJ software.

### Fluorescence quantification and statistical analysis

CDKSENS-Cy3 and Ctrl-Cy5 were complexed individually with CADY2 and codelivered into cells. Live-cell imaging acquisitions were substracted for background signal corresponding to minimal fluorescence levels using Metamorph. Image J was then used for image analysis. Regions of interest (ROI) corresponding to 10–15 cells in which fluorescence appeared homogeneous were designed and the mean grey levels of fluorescence for each channel (Cy3 and Cy5) were quantified within these ROIs. For each experiment, and the mean ratiometric value of CDKSENS-Cy3/Ctrl-Cy5 was calculated from 3–4 different ROIs. Each experiment was performed 3–4 times to assess the robustness of the quantification strategy. All statistical analyses are presented by the mean and standard deviation.

## Supporting Information

Figure S1 **Further in vitro characterization of CDKSENS.** (**A**) Titration of CDKSENS-Cy3 with recombinant GST-CDK2 and GST-Cyclin A (**B**) Titration of CDKSENS-FITC with preformed complexes of CDK2/Cyclin B (K2/B), CDK1/Cyclin A (K1/A) and CDK1/Cyclin B (K1/B).
(TIF)

Figure S2 **Colocalization of CDKSENS, CDKs and Cyclins.** Low magnification of Fig. 3D. Subcellular localization of CDKSENS-Cy3 delivered into HeLa cells with CADY2, and of endogenous CDKs and Cyclins detected by indirect immunofluorescence and imaged by confocal microscopy.
(TIF)

Figure S3 **CADY2-mediated delivery of Ctrl peptide.** Cy3-labelled Ctrl peptide complexed with CADY2 at 1∶40 ratio is efficiently internalized by HeLa cells (lower panels), in contrast to Cy3-labelled Ctrl peptide alone (upper panels).
(TIF)

Table S1 **Average ratiometric quantification of CDKSENS-Cy3/Ctrl-Cy5 fluorescence determined from individual sets of experiments and normalized to the highest mean ratiometric value.**
(TIF)

Movie S1 **Dynamics of CDKSENS-Cy3 in HeLa cells.**
(AVI)
