# Supplementary figures and images for: Fluorescent Peptide Biosensor for Probing the Relative Abundance of Cyclin-Dependent Kinases in Living Cells
Source: PLoS One. 2011 Oct 18;6(10):e26555. doi: 10.1371/journal.pone.0026555 (PMC3196589; doi:10.1371/journal.pone.0026555)

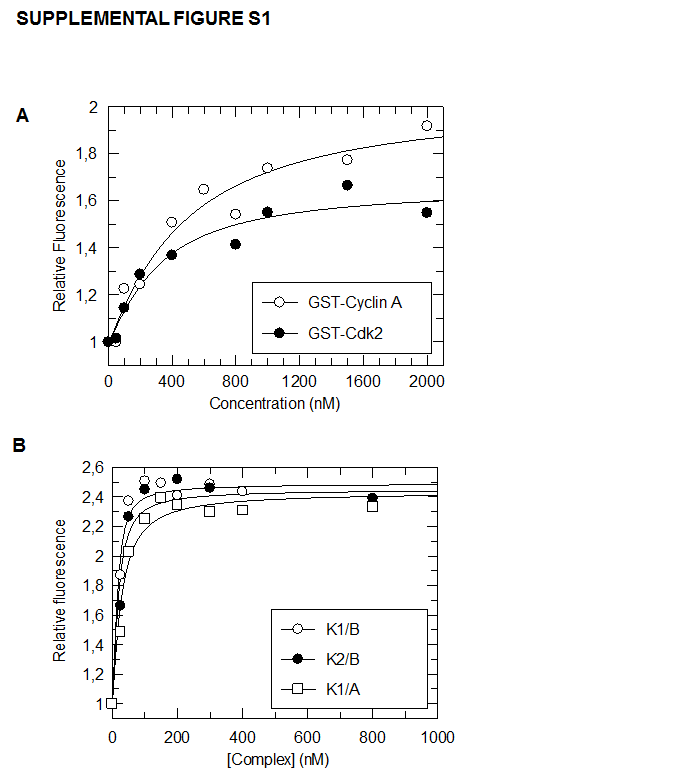

Supplement: Figure S1 — Further in vitro characterization of CDKSENS. (A) Titration of CDKSENS-Cy3 with recombinant GST-CDK2 and GST-Cyclin A (B) Titration of CDKSENS-FITC with preformed complexes of CDK2/Cyclin B (K2/B), CDK1/Cyclin A (K1/A) and CDK1/Cyclin B (K1/B). (TIF) [file pone.0026555.s001.tif]

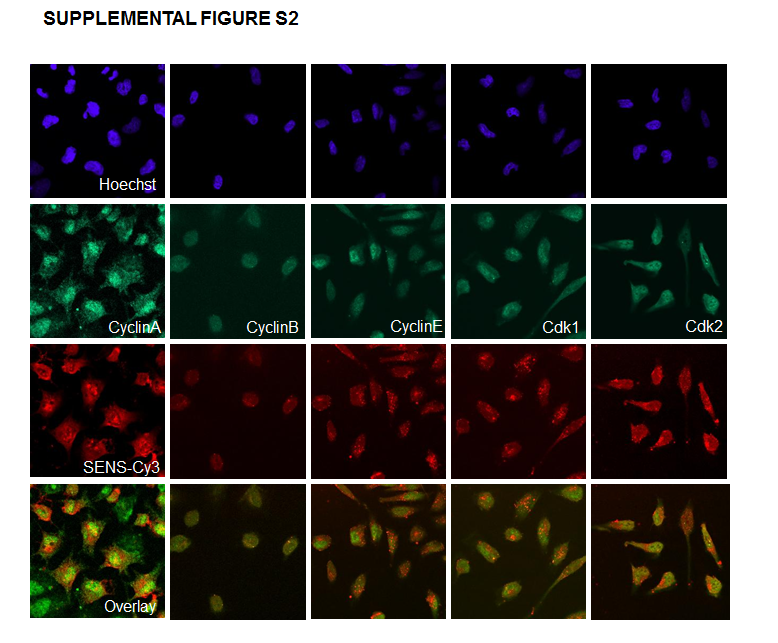

Supplement: Figure S2 — Colocalization of CDKSENS, CDKs and Cyclins. Low magnification of Fig. 3D. Subcellular localization of CDKSENS-Cy3 delivered into HeLa cells with CADY2, and of endogenous CDKs and Cyclins detected by indirect immunofluorescence and imaged by confocal microscopy. (TIF) [file pone.0026555.s002.tif]

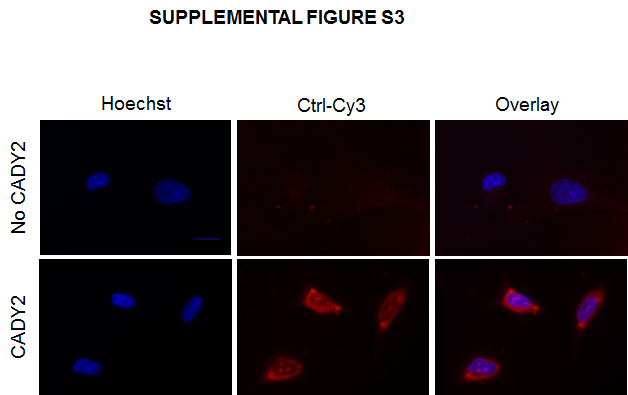

Supplement: Figure S3 — CADY2-mediated delivery of Ctrl peptide. Cy3-labelled Ctrl peptide complexed with CADY2 at 1∶40 ratio is efficiently internalized by HeLa cells (lower panels), in contrast to Cy3-labelled Ctrl peptide alone (upper panels). (TIF) [file pone.0026555.s003.tif]

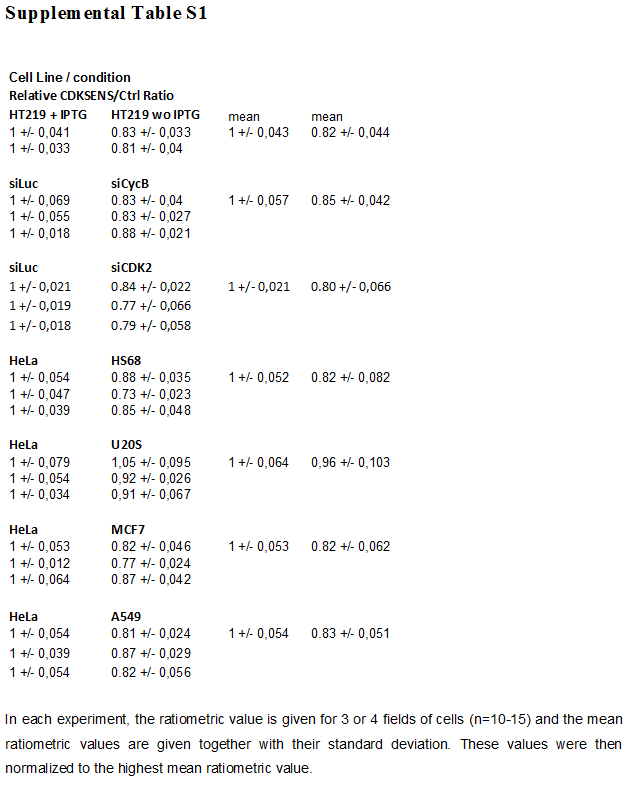

Supplement: Table S1 — Average ratiometric quantification of CDKSENS-Cy3/Ctrl-Cy5 fluorescence determined from individual sets of experiments and normalized to the highest mean ratiometric value. (TIF) [file pone.0026555.s004.tif]
